# EML4-ALK抑制剂在非小细胞肺癌中发生耐药的机制

**DOI:** 10.3779/j.issn.1009-3419.2013.01.09

**Published:** 2013-01-20

**Authors:** 荻 吴, 鸿 于, 佳美 李

**Affiliations:** 1 130012 长春，吉林省肿瘤医院肿瘤内科 Department of Medical Oncology, Jilin Province Tumor Hospital, Changchun 130012, China; 2 130021 长春，吉林大学第一医院肿瘤中心 Tumor Center, First Hospital of Jilin University, Changchun 130021, China; 3 130012 长春，吉林省肿瘤防治研究所细胞生物研究室 Cell Biology Laboratory, Jilin Province Tumor Institute, Changchun 130012, China; 4 130012 长春，吉林大学白求恩医学院临床医学七年制 Clinical Medicine, Norman Bethune College of Medicine, Jilin University, Changchun 130012, China

非小细胞肺癌（non-small cell lung cancer, NSCLC）约占肺癌总数的80%-85%，是最常见的恶性肿瘤之一。继表皮生长因子受体（epithelial growth factor receptor, *EGFR*）基因突变在NSCLC发生过程中的机制逐渐明晰后，NSCLC的靶向治疗已经成为临床实践的热点^[1]^。近年来的研究^[2]^发现了一种新的癌基因，棘皮动物微管相关蛋白样4-间变性淋巴瘤激酶（echinoderm microtubule-associated protein-like 4-anaplastic lymphoma kinase, EML4-ALK）参与了NSCLC的发生过程。以EML4-ALK为靶点的分子靶向药物的应用成为治疗NSCLC的焦点，包括克唑替尼（crizotinib）在内的多种酪氨酸激酶抑制剂（tyrosine kinase inhibitors, TKI）在临床前期研究以及对ALK易位癌症患者的治疗中取得良好的疗效^[3, 4]^。虽然该药在2011年已被FDA批准，但并不是所有的患者均能从治疗中获益，一部分患者经治后疾病进展，产生获得性耐药; 还有很少的一部分患者初治即发生耐药，因此探究TKI耐药机制具有重要的临床意义，阐明其耐药性机制将有利于制定克服临床耐药性发生的新策略^[5]^。本文将详述目前已知的耐药机制。

## 1 ALK激酶区域突变

一个公认的TKI获得性耐药的机制是在激酶域ATP结合位点发生集群性突变^[6, 7]^。Crizotinib的耐药机制研究仍在探索中，从所有经过crizotinib治疗的NSCLC以及炎性肌纤维母细胞瘤（inflammatory myofibroblastic tumor, IMT）患者中证实的几种二次突变都已公开报道^[8, 9]^。2008年，Choi等^[10]^首次报道在临床治疗中发现一例患者持续应用crizotinib后肿瘤继续增长。推测可能发生了基因的二次突变，诱发继发性耐药。研究组利用Sanger测序在73例EML4-ALK阳性患者胸腔积液标本中检测到序列4374G→A和4493C→A两种基因突变，其中，34例（46.6%）呈4374G→A阳性; 11例（15.1%）呈4493C→A阳性。相应序列1156和1196表达的氨基酸发生了改变。其余28例（38.4 %）两个点突变均为阴性。C1156和L1196都位于ALK激酶域，其中C1156位于预测的螺旋αC氨基末端，接近ATP结合区域上部边缘; L1196则位于ATP结合区域底部“gatekeeper”处，这种带着大分子侧链的氨基酸可能会干扰TKI的结合。

2010年，Sasaki等^[9]^也证实L1196突变可能创建了一个空间位阻阻止crizotinib的结合。而F1174L突变可能促进ALK活化构象的产生，从而不利于crizotinib的结合，使其优先结合无效构象ALK。2011年，发现一种新的ALK TKI耐药机制，即在ALK和活化的EGFR信号中的一个二次突变（L1152R），导致在1152位置上的亮氨酸被精氨酸取代^[6]^。值得注意的是这些突变可以发生在同一个肿瘤中，故在制定克服临床耐药的策略时应该充分认识到耐药机制的复杂性^[11]^。

在已经鉴定的各种不同EML4-ALK亚型中，没有任何证据表明特定EML4-ALK亚型获得性耐药的机制会有所不同。此外，L1152R突变与F1174L突变不同，其对另外一个ALK抑制剂TAE684敏感性不同，故需要结构独特的ALK抑制剂克服这种突变。有几种新型抑制剂正在临床前开发阶段。与此同时，Zhang等^[12]^鉴定了在激酶活性部位周围的5个区域的残基突变，除已知的C1156、F1174、L1196和L1152等突变外，还检测到另外两种新的二次突变S1206和I1171。相信随着对*ELM4-ALK*融合基因研究的深入，可能会发现更多不同突变会导致耐药性的发生。

2012年，Doebele等^[13]^通过直接对ALK外显子21-25（编码激酶区域）进行测序，分析了14例患者ALK重组的NSCLC组织，其中11例有分子分析的评估价值，4例（36%）在ALK酪氨酸激酶区域产生二次突变，2例在相应区域编码L1196M替代氨基酸。该基因在经典的“gatekeeper”基因水平上与T315I和T790M同源; 有2例在ALK编码区域发生一种新的二次基因突变（G1269A），位于ALK的ATP结合区末端，原来的甘氨酸被较大的丙氨酸取代，形成一定的空间位阻阻碍crizotinib的结合。体外实验证明，与野生型相比，G1269A能够促进ALK永久性磷酸化，激活下游效应器，发生crizotinib耐药。

同在2012年，Katayama等^[14]^探讨了经crizotinib治疗后的耐药属于继发性耐药还是原发性耐药的问题。针对18例crizotinib经治耐药患者的样本，他们进行了治疗基线与耐药后组织总核酸测序以及ALK激酶域的标准Sanger测序。在相应crizotinib耐药样本中未发现其治疗前基线组织存在*ALK*突变。使用等位基因特异的PCR检测法进行再确认，所得结果与前次实验一致。这表明crizotinib的耐药突变在治疗前的肿瘤组织中并不普遍。通过对外显子20-28对应的ALK酪氨酸激酶域测序。在18例crizotinib耐药患者中，发现4种耐药突变：3种错义突变(L1196M、G1202R和S1206Y）以及1种氨基酸（苏氨酸）插入突变(1151Tins）。L1196M替代氨基酸突变既往已被公开报道。G1202R突变与伊马替尼耐药的BCR-ABL突变（G340W）类似。其它两个突变（1151T插入和S1206Y）似乎为ALK和crizotinib耐药所特有。G1202R和S1206Y都位于邻接crizotinib结合位点激酶结构域的溶剂暴露区域。推测1151Tins残基位于α螺旋C的N末端环路远端，此位置与crizotinib结合位点并不相邻。推测该突变可能会影响ALK对ATP的亲和力，导致高水平的crizotinib耐药。

通过对以上突变编码的氨基酸被映射到ALK激酶区域晶体结构的研究^[15]^，发现该系列突变出现在或者远离ATP和crizotinib的结合口袋区域的狭长凹陷处。2012 ASCO报道（Abstract No: 7504）Doebele等收集30例ALK阳性的经crizotinib治疗进展后的NSCLC病例，19例取得活检组织，15例可最终评价分子学指标。结果发现6例（40%）发生ALK激酶域继发突变，并新发现了F1174C和D1203N突变; 2例（其中1例有耐药突变）出现新的*ALK*基因拷贝数增益（copy number gain, CNG）; 4例出现其它基因位点的突变（1例*EGFR*突变; 3例*KRAS*突变），伴或不伴*ALK*基因融合; 1例肿瘤进展的样本缺乏*ALK*融合基因; 3例ALK阳性样本没有明确的耐药机制。该研究进一步证明了耐药机制发生的复杂性和临床治疗克服耐药面临的诸多挑战^[16]^。

随着研究的扩展和深入，更多的*ALK*融合基因的继发突变被发现。在NSCLC中*ALK*融合基因的发生率较低，并且还分为多种亚型，这给临床检测鉴定带来了困难。不同亚型与继发突变之间有何相关性，多个继发突变位点之间又有什么联系，很多问题有待深入研究。尤其是对于中国的患者，尚需精确检测并大规模的分析*ALK*融合的发生率和不同*ALK*融合基因亚型的发生频率及其与EGFR等其它分子靶点之间的关系。

## 2 信号传导旁路的激活

ALK是一个高度保守的受体酪氨酸激酶（receptor tyrosine kinase, RTK），作为核磷蛋白（nucleophosmin, NPM）融合物，在1994年首次在间变性大细胞淋巴瘤（Anaplastic Large Cell Lymphoma, ALCL）中发现^[17]^。ALK有三个结构域：胞外配体结合结构域，跨膜区和胞内酪氨酸激酶域。就同源性来说，ALK与白细胞酪氨酸激酶（leukocyte tyrosine kinase, LTK）是最相似的，都属于胰岛素受体超家族。在生理条件下结合配体诱导的ALK二聚体可导致反式磷酸化和激酶的活化^[18]^。此外，不同于原来的ALK定位于质膜，ALK融合蛋白的大部分分布到细胞质中，这种在细胞定位上的不同可能导致ALK激活机制的改变。

ALK的关键下游效应信号比上游信号的激活更为人们所了解，包括Ras/MEK/ERK、PI3K/AKT和JAK3-STAT3信号转导通路^[19, 20]^。在一般情况下，Ras/MEK/ERK是重要的细胞增殖信号传导通路，而PI3K/AKT和JAK3-STAT3通路对于细胞存活和细胞骨架的变化是很重要的。药理学抑制就是通过使用EML4-ALK TKI使Ras/MEK/ERK和PI3K/AKT通路分子表达下调，从而诱导细胞凋亡^[21]^。小分子TKI crizotinib（PF02341066）作为一种强效的ALK抑制剂^[22]^，与NPM-ALK阳性的ALCL细胞G1-S期的细胞周期阻滞相关，抑制ALK磷酸化和信号转导，并诱导细胞凋亡。在细胞和转基因小鼠模型的体内外研究中，EML4-ALK是一种强效致癌的“驱动突变”^[21, 23]^。含有这种基因重组的癌细胞变得依赖或“沉溺”于ALK，对ALK激酶抑制是高度敏感的^[24]^。然而，在ALK抑制剂耐药模型中，即使持续使用酪氨酸激酶抑制剂，Ras/MEK/ERK以及PI3K/AKT传导通路仍都被再次激活^[18]^。即ALK下游信号传导旁路的激活会绕过抑制剂作用的原始靶点，成为ALK抑制剂耐药性产生的原因之一^[25]^。

在2012 ASCO的教育专场中，Camidge也指出ALK TKI的耐药机制可能包括其它旁路途径的活化，如EGFR、KIT受体的活化。可见，ALK TKI的耐药机制是多样化的，需要积累样本开展大规模的综合分析，为最终克服耐药提供方法和数据。

Takezawa等^[26]^为了研究EML4-ALK致癌的功能，建立了非转化的小鼠成纤维细胞系（NIH 3T3）。这些细胞可以稳定表达EML4-ALK的亚型1或3（3T3/EAV1和3T3/EAV3细胞）。研究中发现，与3T3-模拟细胞相比，3T3/EAV1和3T3/EAV3细胞表达磷酸化的ERK和STAT3水平都明显增加，而EML4-ALK的表达并不影响3T3-模拟细胞AKT的磷酸化水平。他们对3T3/EAV1和3T3/EAV3细胞使用ALK的siRNA进行EML4-ALK RNA干扰（RNA interference, RNAi），明显抑制ERK和STAT3的磷酸化，但对AKT无明显影响。这些数据表明，EML4-ALK亚型1或3可以激活ERK和STAT3信号通路，而不是PI3K/AKT信号传导通路。进一步发现，在所有3种细胞系中新型酪氨酸激酶抑制剂TAE684抑制ERK和STAT3的激活，同样不影响AKT。这表明ALK抑制剂诱导EML4-ALK阳性肺癌细胞的生长抑制和凋亡，伴随这些影响的不是PI3K/AKT信号通路而是ERK及STAT3信号转导通路。ALK抑制剂可以明显抑制ERK及STAT3信号转导通路，PI3K/AKT信号通路则可能不受影响或者影响较小，可以继续传递信号，成为耐药性产生的原因之一。

最近的研究^[19]^表明，sonic hedgehog信号传导通路（SHH/GLI1）也在ALK阳性ALCL内被激活。SHH/GLI1是由于*SHH*基因扩增，其实是*NPM-ALK*基因通过激活PI3K/AKT信号通路进一步介导该通路并产生稳定的GLI1蛋白。这一AKT旁路的激活也可能是导致耐药性的原因。

Katayama等^[27]^培育的H3122 CR3细胞对ALK抑制剂和HSP90抑制剂均耐药。Crizotinib作用于该细胞系后，其抑制磷酸化ALK的水平与敏感性亲代细胞程度相同。然而，尽管受ALK抑制，AKT和ERK仍保持活化。这表明AKT和ERK信号传导途径的活化是由ALK以外的调节器维持的。磷酸化受体酪氨酸激酶微列阵分析表明，H3122 CR3细胞与亲代细胞相比，在crizotinib治疗前后都含有更高水平的磷酸化EGFR和磷酸化ERBB3。另外，Real Time RT-PCR分析表明，在耐药细胞中，EGFR mRNA、EGFR配体双调蛋白和ERBB3配体NRG1有所上调，推测H3122 CR3细胞内EGFR活化可能是由于上调受体本身以及两个配体导致持久性ALK非依赖的下游信号转导通路的活化。

Sasaki等^[6]^的研究同时表明，EGFR信号通路的激活作为旁路的信号传导机制，也导致ALK抑制剂耐药，同时抑制EGFR和ALK的方法对所有耐药模型的治疗有效。

上述研究结果提示我们为了克服crizotinib耐药，可以将其与其它的信号通路抑制剂联合使用（如培美曲塞、EGFR抑制剂或HSP90抑制剂），或者与其它的ALK抑制剂联合使用。

## 3 *ALK*融合基因拷贝数目增益

拷贝数目的增益定义为与预处理样本相比，治疗后样本每个细胞内重组基因数目有2倍以上的增加。最近的研究^[27]^表明*ALK*融合基因拷贝数目的增益与crizotinib耐药性相关。通过荧光原位杂交技术（fluorescent in situ hybridization, FISH）可以检测crizotinib治疗前后每个细胞基因重组ALK拷贝的含量^[28]^。在评估的90例样本中30例接受了crizotinib治疗，检测结果表明ALK阳性细胞有更高的拷贝数（*r*=0.743, *P* < 0.000, 1）。在ALK阳性的肿瘤中，阳性细胞的百分比和拷贝数都是预测ALK抑制剂的治疗作用的有效指标。Katayama等^[14]^报道了18例获得crizotinib耐药的肺癌患者中有1/4伴有*ALK*融合基因的扩增，而且在发生耐药的患者中多种耐药机制并存。Doebele等^[13]^的研究中有2例患者（18 %）出现明显的ALK重组基因增益。这些结果都表明，*ALK*融合基因的大量扩增可能导致ALK抑制剂耐药性的产生。

## 4 其它机制

在Doebele等^[13]^的研究中还有2例患者显示为*KRAS*基因突变，其中1例并没有明显的证据表明其含有永久性的*ALK*基因重组; 1例患者与基线样本相比变为*ALK*融合基因阴性，没有明确的启动机制; 2例患者始终保持ALK阳性，但没有明确其耐药机制。因此考虑独立致癌驱动因子等因素与耐药性的产生有关。综上所述，ALK-TKI分子靶向治疗不可避免的产生耐药问题，使得其对患者的治疗疗效逐渐降低。其耐药机制复杂，主要包括：①ALK激酶区域的二次突变（不同的突变基因导致的耐药机制还有所不同，进一步的多元耐药机制还需要深层的研究）; ②信号传导旁路的激活，使得信号传导绕过抑制剂作用的原始靶点，产生耐药性; ③*ALK*融合基因增益; ④产生非依赖性的致癌因子等机制都与其耐药性相关。有很多机制还处于未知状态，有待于进一步的研究。目前已知的ALK抑制剂发生耐药的几种机制见图 1。

**1:**
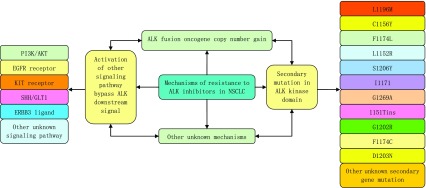
ALK抑制剂在非小细胞肺癌中发生耐药的不同机制 Multiple mechanisms of resistance to ALK inhibitors in non-small cell lung cancer (NSCLC)

## 5 ALK抑制剂在其它肿瘤治疗中的应用

*ALK*基因异常主要发生在间变性大细胞淋巴瘤、神经母细胞瘤、炎性肌纤维母细胞瘤和NSCLC中。在一个由多中心儿童肿瘤组织开展的Ⅰ期临床试验中，参与的儿童通过服用crizotinib获得了缓解，而且副作用很小。2012 ASCO报道了这项研究发现。8例患有ALCL的儿童中，有7例完全缓解; 7例有*ALK*基因易位的IMT患者，1例微效，1例部分缓解; 35例神经母细胞瘤患者，27例可评价疗效。8/27例患者有*ALK*基因突变：1例治疗后完全缓解并维持超过6个月，1例微效。2例病变稳定超过8个疗程。1例NSCLC患者部分缓解。Butrynski等^[8]^报道1例ALK易位的IMT患者对crizotinib部分反应。患者接受crizotinib治疗8个月后无疾病进展，肿瘤切除术后19个月无复发。Lennerz等^[29]^报道2例Met扩增胃食管癌患者对crizotinib敏感。治疗后肿瘤缩小（-30%和-16%）并持续了3.7个月和3.5个月。Gambacorti-Passerini等^[30]^报道2例复发的ALK阳性ALCL患者crizotinib治疗后1周显示了临床改善，完全应答分别持续了6个月和15个月。

ALK抑制剂在其它肿瘤中的耐药机制报道较少。Sasaki等^[9]^报道携带RANBP2-ALK易位的IMT患者使用crizotinib治疗后疾病进展是由ALK的F1174L突变所致。一个神经母细胞瘤细胞系SK-N-SH，在*ALK*基因中含有F1174L突变，在用TAE684处理后发生耐药伴随STAT3磷酸化水平升高^[31]^。这也提示NSCLC中发生ALK抑制剂耐药的复杂性和特殊性。

## 6 发展前景

在了解有关的耐药机制后最关注的是如何根据不同的耐药机制克服耐药问题。Katayama等^[27]^证实另一种强效ALK抑制剂AP26113，可以克服crizotinib耐药。而Li等^[32]^在研究中也使用了一个强效有力并且有选择性的ALK小分子抑制剂-NPV-TAE684，以评估EML4-ALK在NSCLC中的致癌作用。他们的实验结果表明TAE684可以在*EML4-ALK*融合基因阳性的NSCLC模型中抑制细胞增殖、诱导细胞周期阻滞、凋亡和肿瘤消退。他们用微阵列分析方法，有针对性地开展对TAE684治疗后H2228 NSCLC移植瘤模型基因表达途径变化的研究，并确定了*EML4-ALK*抑制的基因印记（基因印记表示1210已知的人类基因，以及最佳的生物过程表代表这些基因的细胞周期、DNA合成、细胞增殖和细胞死亡）。他们还比较了TAE684和crizotinib的疗效，虽然crizotinib能够减少H2228和H3122细胞的生存率，但与TAE684相比不太有效：H2228和H3122相对的crizotinib IC_50_值分别为871 nmol/L和1, 551 nmol/L，而TAE684对应的IC_50_值为16 nmol/L（H2228）和44 nmol/L（H3122）。证明了TAE684是一个更有力的EML4-ALK抑制剂，在crizotinib耐药性NSCLC的治疗中可能取得更好的疗效，在克服耐药的实验研究进程中起重要的作用。

2012年欧洲肿瘤内科学会报道了一些新的ALK抑制剂的研发，对crizotinib无效的患者也有活性。一项小样本的研究结果（Abstract No: 440_O）探讨了LDK治疗56例ALK阳性的肿瘤患者的疗效。在接受crizotinb治疗后疾病进展的患者中，这个药物疗效明显; 另一个ALK抑制剂CH5424802^[33]^也对ALK阳性的NSCLC患者显示出有临床意义的抗肿瘤活性(Abstract No: 441_O); 此外，HSP90抑制剂也代表一种新型的治疗策略^[34, 35]^。其中AUY922对crizotinb治疗后疾病进展的患者或EGFR抑制剂（吉非替尼或厄洛替尼）治疗后进展的EGFR阳性患者均有治疗作用(Abstract No: 438_O)。一种HSP90抑制剂17-烯丙基氨基-17-去甲氧基格尔德霉素（17-AAG）能够抑制四种突变形式的EML4-ALK，类似于其对野生型EML4-ALK的效力。17-AAG降低表达野生型或突变型EML4-ALK的Ba/F3细胞株的磷酸化ALK和所有ALK蛋白的表达，克服crizotinib耐药介导的二次ALK突变，尤其是像1151Tins这类经检测对所有ALK抑制剂高水平耐药的突变^[27]^。

已经发现ALK的二次突变可同时伴有*EGFR*突变，说明crizotinib耐药可能同时由多种耐药机制共同诱导。因此，融合性的二代新型抗肿瘤药物的研发也势在必行。对于EGFR TKI耐药的研究也给出很多启示，采取全身治疗（原靶向治疗药物、新靶向治疗药物和细胞毒性药物等）和局部治疗（手术、放疗和各种介入治疗）的联合以克服耐药性，从而尽可能有效的治疗癌症。此外，多靶点酪氨酸激酶抑制剂可以多途径干扰肿瘤细胞的发生和发展，为TKI耐药患者带来福音。但目前大部分多靶点酪氨酸激酶抑制剂仍处于临床试验阶段，其最佳适用人群尚需进一步研究。

已经进行的第一项crizotinib的Ⅲ期临床研究（PROFILE 1007）显示crizotinib带来的改善是非常明显的，疾病进展风险降低50%。在欢欣鼓舞的同时更加迫切的需要解决目前存在的诸多问题。ALK抑制剂发生耐药性的频率，不同耐药机制的发生频率，各种耐药机制之间的相互作用关系，以及ALK抑制剂耐药与其他TKI之间的关系尚待研究。Heuckmann等^[36]^已证明不同的*ALK*融合基因和EML4-ALK亚型对crizotinib和TAE684的敏感性不同，其机制与蛋白表达的稳定性相关。有趣的是，它们对HSP90抑制的敏感性也不同，而且这种不同与它们对ALK抑制剂的敏感性也是有差异的。二者合用效果更佳。这些结果提示需要精确检测ALK的基因型并探讨研究ALK抑制剂的组合应用策略。此外，多种突变共存的患者是否可以使用融合性新型抗肿瘤药物治疗，单药应用时通过药物假期和再次给药的方式能否有效的克服耐药，新研制开发的药物在已经发生耐药的患者中的治疗效果和安全性等问题还有待实践验证。

## References

[1] Heist RS, Engelman JA (2012). SnapShot: non-small cell lung cancer. Cancer Cell.

[2] Soda M, Choi YL, Enomoto M (2007). Identification of the transforming EML4-ALK fusion gene in non-small cell lung cancer. Nature.

[3] Hallberg B, Palmer RH (2011). ALK and NSCLC: Targeted therapy with ALK inhibitors. F1000 Med Rep.

[4] Okamoto I, Nakagawa K (2012). Echinoderm microtubule-associated protein-like 4-anaplastic lymphoma kinase-targeted therapy for advanced non-small cell lung cancer: molecular and clinical aspects. Cancer Sci.

[5] Camidge DR, Doebele RC (2012). Treating ALK-positive lung cancer--early successes and future challenges. Nat Rev Clin Oncol.

[6] Sasaki T, Koivunen J, Ogino A (2011). A novel ALK secondary mutation and EGFR signaling cause resistance to ALK kinase inhibitors. Cancer Res.

[7] Pao W, Miller VA, Politi KA (2005). Acquired resistance of lung adenocarcinomas to gefitinib or erlotinib is associated with a second mutation in the EGFR kinase domain. PLoS Med.

[8] Butrynski JE, D'Adamo DR, Hornick JL (2010). Crizotinib in ALK-rearranged inflammatory myofibroblastic tumor. N Engl J Med.

[9] Sasaki T, Okuda K, Zheng W (2010). The neuroblastoma-associated *F1174L* ALK mutation causes resistance to an ALK kinase inhibitor in ALK-translocated cancers. Cancer Res.

[10] Choi YL, Soda M, Yamashita Y (2010). *EML4-ALK* mutations in lung cancer that confer resistance to ALK inhibitors. N Engl J Med.

[11] Sasaki T, Rodig SJ, Chirieac LR (2010). The biology and treatment of EML4-ALK non-small cell lung cancer. Eur J Cancer.

[12] Zhang S, Wang F, Keats J (2011). Crizotinib-resistant mutants of EML4-ALK identified through an accelerated mutagenesis screen. Chem Biol Drug Des.

[13] Doebele RC, Pilling AB, Aisner DL (2012). Mechanisms of resistance to crizotinib in patients with *ALK* gene rearranged non-small cell lung cancer. Clin Cancer Res.

[14] Katayama R, Shaw AT, Khan TM (2012). Mechanisms of acquired crizotinib resistance in ALK-rearranged lung Cancers. Sci Transl Med.

[15] Lee CC, Jia Y, Li N (2010). Crystal structure of the ALK (anaplastic lymphoma kinase) catalytic domain. Biochem J.

[16] Sasaki T, Jänne PA (2011). New strategies for treatment of ALK-rearranged non-small cell lung cancers. Clin Cancer Res.

[17] Morris SW, Kirstein MN, Valentine MB (1994). Fusion of a kinase gene, *ALK*, to a nucleolar protein gene, *NPM*, in non-Hodgkin's lymphoma. Science.

[18] Shaw AT, Solomon B (2011). Targeting anaplastic lymphoma kinase in lung cancer. Clin Cancer Res.

[19] Mossé YP, Wood A, Maris JM (2009). Inhibition of ALK signaling for cancer therapy. Clin Cancer Res.

[20] Chiarle R, Simmons WJ, Cai H (2005). Stat3 is required for ALK-mediated lymphoma genesis and provides a possible therapeutic target. Nat Med.

[21] Koivunen JP, Mermel C, Zejnullahu K (2008). *EML4-ALK* fusion gene and efficacy of an ALK kinase inhibitor in lung cancer. Clin Cancer Res.

[22] Christensen JG, Zou HY, Arango ME (2007). Cytoreductive antitumor activity of PF-2341066, a novel inhibitor of anaplastic lymphoma kinase and c-Met, in experimental models of anaplastic large-cell lymphoma. Mol Cancer Ther.

[23] Soda M, Takada S, Takeuchi K (2008). A mouse model for EML4-ALK-positive lung cancer. Proc Natl Acad Sci USA.

[24] McDermott U, Iafrate AJ, Gray NS (2008). Genomic alterations of anaplastic lymphoma kinase may sensitize tumors to anaplastic lymphoma kinase inhibitors. Cancer Res.

[25] Qi J, McTigue MA, Rogers A (2011). Multiple mutations and bypass mechanisms can contribute to development of acquired resistance to MET Inhibitors. Cancer Res.

[26] Takezawa K, Okamoto I, Nishio K (2011). Role of ERK-BIM and STAT3-survivin signaling pathways in ALK inhibitor-induced apoptosis in EML4-ALK-Positive Lung Cancer. Clin Cancer Res.

[27] Katayama R, Khan TM, Benes C (2011). Therapeutic strategies to overcome crizotinib resistance in non-small cell lung cancers harboring the fusion oncogene EML4-ALK. Proc Natl Acad Sci USA.

[28] Camidge DR, Theodoro M, Maxson DA (2012). Correlations between the percentage of tumor cells showing an ALK gene rearrangement, *ALK* signal copy number and response to crizotinib therapy in ALK FISH positive non-small cell lung cancer. Cancer.

[29] Lennerz JK, Kwak EL, Ackerman A (2011). MET amplification identifies a small and aggressive subgroup of esophagogastric adenocarcinoma with evidence of responsiveness to crizotinib. J Clin Oncol.

[30] Gambacorti-Passerini C, Messa C, Pogliani EM (2011). Crizotinib in anaplastic large-cell lymphoma. N Engl J Med.

[31] Yan X, Kennedy CR, Tilkens SB (2011). Cooperative cross-talk between neuroblastoma subtypes confers resistance to anaplastic lymphoma kinase inhibition. Genes Cancer.

[32] Li Y, Ye X, Liu J (2011). Evaluation of EML4-ALK fusion proteins in non-small cell lung cancer using small molecule inhibitors. Neoplasia.

[33] Sakamoto H, Tsukaguchi T, Hiroshima S (2011). CH5424802, a selective ALK inhibitor capable of blocking the resistant gatekeeper mutant. Cancer Cell.

[34] Sequist LV, Gettinger S, Senzer NN (2010). Activity of IPI-504, a novel heat-shock protein 90 inhibitor, in patients with molecularly defined non-small-cell lung cancer. J Clin Oncol.

[35] Chen Z, Sasaki T, Tan X (2010). Inhibition of ALK, PI3K/MEK, and HSP90 in murine lung adenocarcinoma induced by *EML4-ALK* fusion oncogene. Cancer Res.

[36] Heuckmann JM, Balke-Want H, Malchers F (2012). Differential protein stability and ALK inhibitor sensitivity of *EML4-ALK* fusion variants. Clin Cancer Res.

